# Development of a Synthesized Gene Unique to Lumpy Skin Disease Virus and Its Application in Serological Differentiation of Naturally Infected from Vaccinated Cattle with Attenuated Goat Pox Vaccine

**DOI:** 10.1155/2024/7800855

**Published:** 2024-06-10

**Authors:** Xinwei Yuan, Haoyun Zhang, Yu Wang, Di Wu, Ihsanullah Shirani, Yingyu Chen, Jianguo Chen, Xi Chen, Lei Zhang, Huanchun Chen, Changmin Hu, Aizhen Guo

**Affiliations:** ^1^ National Key Laboratory of Agricultural Microbiology College of Veterinary Medicine Huazhong Agricultural University Wuhan 430070China; ^2^ Hubei Hongshan Laboratory Wuhan 430070China; ^3^ The Cooperative Innovation Center for Sustainable Pig Production Huazhong Agricultural University Wuhan 430070China; ^4^ Hubei International Scientific and Technological Cooperation Base of Veterinary Epidemiology Wuhan 430070China

## Abstract

Lumpy skin disease (LSD) is an important infectious disease caused by lumpy skin disease virus (LSDV) in bovine. LSDV, sheep pox virus (SPPV), and goat pox virus (GTPV) from the same genus *Capripoxvirus* (CaPV) of the *Poxviridae* family exhibit a nucleotide sequence similarity of up to 97%. Therefore, attenuated vaccines of GTPV and SPPV are often used to vaccinate cattle against LSD. However, available serological testing methods cannot accurately differentiate cattle vaccinated with GTPV from those infected with LSDV, posing a significant risk for disease spread. In this study, we developed a synthesized gene unique to LSDV as a differential antigen to detect serum antibodies specific to LSDV and differentiate naturally infected from vaccinated animals (DIVA). We used it for an in-house indirect enzyme-linked immunosorbent assay (iELISA), and no cross-reaction with positive sera for bovine viral diarrhea virus (BVDV), infectious bovine rhinotracheitis virus (IBRV), *Mycobacterium bovis* (*M. Tb*), *Pasteurella multocida* (*P. multocida*), and *Mycoplasma bovis* (*M*. *bovis*). The cut-off value (*S*/*P*%) was 30% for in-house iELISA. The corresponding diagnostic specificity was 100% (95% CI: 88.43–100), and the diagnostic sensitivity was 93.3% (95% CI: 77.93–99.18). The intra-assay coefficient of variation (CV) ranged from 1.08% to 4.11%, and the interassay CV was 0.00%–8.90%. Furthermore, 200 clinical serum samples were examined, in the vaccinated herd, there were no positive samples (0/141) indicating the strong differentiation ability of this method. On the other hand, in the infected herds, the overall positivity was 33.90% (20/59) (95% CI: 22.08–47.39). In summary, a valuable synthesized protein unique to LSDV was developed and showed a promising application in an iELISA with high specificity and sensitivity in differentiating cattle infected with LSDV from those vaccinated with GTPV.

## 1. Introduction

Lumpy skin disease (LSD) caused by lumpy skin disease virus (LSDV) is a highly infectious disease in bovine. LSDV exhibits high host specificity, primarily infecting cattle and water buffalo [1]. Clinically, LSD manifests as generalized skin lumps on cattle [2], resulting in various detrimental consequences on productivity and animal welfare, such as reduced milk production, delayed growth, abortion for pregnant cows, and temporary or prolonged sterility [3, 4]. The incidence rate of LSD can vary from 5% to 45%, with a mortality rate of approximately 5% [5]. LSDV can infect beef, dairy, and yak, and leading to significant economic losses in the cattle industry [6, 7]. LSD first outbroke in August 2019 in Xinjiang, China, and has successively been reported in 17 provinces, causing severe economic losses [8].

LSDV belongs to the *Capripoxvirus* (CaPV) genus of the *Poxviridae* family, a double-stranded DNA virus with a capsule membrane [9, 10]. LSDV has a relatively large genome comprising 156 putative genes and shares high nucleotide sequence similarity (97%) with other viruses in the same genus, such as sheep pox virus (SPPV) and goat pox virus (GTPV), thus resulting in considerable antigenic similarity [11, 12, 13]. Consequently, the live attenuated goat pox and sheep pox vaccines are widely used to prevent LSD outbreaks [9, 10].

Traditionally, LSD diagnosis primarily relies on clinical signs, making the identification of mild or subclinical cases challenging [14]. World Organization for Animal Health (WOAH) has recommended virus neutralization test (VNT) as the gold standard for detecting serum antibodies against LSDV. In addition, alternative serological techniques such as enzyme-linked immunosorbent assay (ELISA) are also available [15]. At present, LSDV serum antibody detection methods mainly include VNT and ELISA. Although VNT is a valuable method for measuring the titers of neutralizing antibodies postinfection and postvaccination, it is more time-consuming than ELISA, susceptible to cell contamination, and holds inherent biases arising from operator's subjective observation and judgment [16]. Furthermore, European Food Safety Authority (EFSA) has reported that serum neutralizing antibodies may not be detectable in cattle with mild or asymptomatic LSDV infection, highlighting the potential challenge in accurately assessing serum neutralizing antibody levels in LSDV-infected cattle [17]. Due to cross-reactivity with other poxviruses, available VNT and ELISA methods cannot differentiate LSDV serum antibodies. Additionally, vaccination with goat pox vaccine may lead to potential side effects such as the formation of small lumps on the skin [18, 19]. Therefore, it is imperative to explore more differentiation diagnostic targets with high specificity for LSD control and eradication efforts [20, 21]. Since live attenuated goat pox vaccine is extensively utilized in China for LSD prevention, there is an urgent need to develop an ELISA capable of distinguishing serum antibodies in cattle infected with LSDV from those vaccinated with live attenuated GTPV.

This study was aimed to design a differential diagnostic target and establish a differentiating infected from vaccinated animals (DIVA) method for LSD. We synthesized a gene *rLSDV-gap*-based on identified six unique fragments of the nucleotide sequences in the protein-coding region of LSDV and transformed this gene into *E. coli* BL21 (DE3) to induce protein expression. This protein presented a good diagnostic specificity and sensitivity when used in iELISA reaction systems, providing a valuable diagnostic tool for preventing and controlling LSD in cattle.

## 2. Materials and Methods

### 2.1. Virus Strains

The study analyzed the nucleotide sequences of two virus stains including LSDV strain China/GD01/2020 (GenBank NO: MW355944.1) and goat pox virus AV41 strain (GenBank NO: MH381810.1).

### 2.2. Serum Samples

Thirty reference serum samples were collected from vaccinated cattle 60 days after being immunized with a fivefold dose of attenuated goat pox vaccine via intradermal injection and confirmed to be positive with VNT and ID Screen® Capripox Double Antigen Multi-species test kit (ID-Vet, Grabels, France), and the results were shown in Tables S1 and S3. These vaccinated serum samples were then utilized to determine the cut-off value and used to evaluate the diagnostic specificity of rLSDV-gap-based iELISA. Besides, 10 vaccinated serum samples identified as positive and high antibody-level by VNT and ID Screen® Capripox Double Antigen Multi-species test kit were used to evaluate the differentiating and diagnostic ability of rLSDV-gap-based iELISA.

In addition, 30 references serum samples of infected cattle in China clinically diagnosed as LSD was collected and serum antibodies against LSDV were confirmed positive by both VNT and ID Screen® Capripox Double Antigen Multi-species test kit (ID-Vet, Grabels, France), and the results were shown in Tables S2 and S4. These sera were used to assess the diagnostic sensitivity of rLSDV-gap-based iELISA.

The positive serum samples of LSDV-unrelated common pathogens of cattle with known backgrounds were used to evaluate the analytical specificity of rLSDV-gap-based iELISA, including bovine viral diarrheal virus (BVDV), infectious bovine rhinotracheitis virus (IBRV), *Mycobacterium bovis* (*M*. *tb*), *Pasteurella multocida* (*P. multocida*), and *Mycoplasma bovis* (*M*. *bovis*).

Furthermore, 200 clinical serum samples with relevant background of natural infection or vaccination were examined for clinical application of rLSDV-gap-based iELISA. Forty serum samples were collected from an infected with LSDV yak farm in Yushu City, Qinghai Province, China, 19 serum samples came from an infected beef cattle farm with LSDV in Daye City, Hubei Province, and 141 serum samples were collected from a beef cattle farm vaccinated with attenuated GTPV vaccine in Zhijiang City, Hubei Province, China. All serum samples were stored at −20°C until used.

### 2.3. Design of LSDV Antigen

To distinguish serum antibodies against LSDV or GTPV, a specific protein termed as “rLSDV-gap” was designed using six unique fragments of the nucleotide sequences in LSDV as the coating antigen (Table 1). We used SnapGene 4.2.4 (Dotmatics, Boston, MA, USA) to compare the nucleotide sequences of protein-coding region between LSDV strains China/GD01/2020 (GenBank NO: MW355944.1) and goat pox virus AV41 strain (GenBank NO: MH381810.1). The comparative analysis identified unique nucleotide sequences in the protein-coding region of LSDV. As a result, six unique fragments of the nucleotide sequences in LSDV were obtained. Further, the nucleotide sequence of *rLSDV-gap* was aligned by Blast with publicized 100 sequences in GenBank, and the results showed that the nucleotide sequence of *rLSDV-gap* was highly conservative, exhibiting 99.08% similarity to that of other isolated strains (Figure S1(a)). In addition, there was no similarity in the nucleotide sequence between synthesized *rLSDV-gap* and bovine popular stomatitis virus (GenBank accession NO: NC_005337.1), a member of parapox virus genus, *Poxviridae* family (Figure S1(b)). Then, these six fragments were concatenated by five flexible peptides (GSGS linker) to synthesize a novel differential diagnosis antigen with the length of each nucleotide fragment being at least 24 bp. Subsequently, codon optimization was performed. The complete nucleotide sequence of synthesized *rLSDV-gap* had a full length of 381 bp.

### 2.4. Production of Recombinant Antigen

For the expression of rLSDV-gap protein, *E. coli* BL21 (DE3) cells harboring the pET-28a expression vector were cultured in 500 mL of LB medium containing 50 mg/mL kanamycin at 37°C with continuous shaking until it reached the midlogarithmic growth phase, as indicated by an OD_600_ value ranging from 0.6 to 0.8. Subsequently, the expression of rLSDV-gap protein was induced by adding isopropyl-*β*-D-thiogalactoside (IPTG) at a final concentration of 0.5 mM at 37°C for 5 hr. The pET-28a (+) blank vector was transformed into *E. coli* BL21 (DE3) cells as a negative control. The positive bacterial cells were harvested, resuspended in PBS (0.01 M, pH 7.4), and crushed using a high-pressure crusher (Life Technologies, Carlsbad, USA). Subsequently, the inclusion body containing rLSDV-gap protein was centrifuged at 12,000 rev/min for 10 min, washed in buffer (containing 20 mM Tris, 2 mM EDTA, 1%Triton X-100, and 1 M urea, pH 8.5) for 5 min at 25°C, centrifuged again at 12,000 rev/min for 15 min, resuspended in binding buffer (20 mM Tris, 2 mM EDTA, and 6 M urea, pH 9.0), and incubated for 12 hr at 4°C with shaking. Afterward, the supernatant was collected by centrifuging at 12,000 rev/min for 15 min. rLSDV-gap protein in the supernatant was eluted with different concentrations of imidazole using *Proteinlso*® Ni-NTA Resin His Bind Purification Filler according to the manufacturer's instruction (TransGen, Beijing, China). The eluate was collected, and the purified rLSDV-gap protein was put into dialysis bag and dialyzed with a buffer containing 20 mM Tris-HCl (pH 8.0), 40 mM NaCl, 1 mM MgCl_2_, 0.8% (v/v) glycerin, and 1 mM DTT to remove imidazole. Further, the purified protein was analyzed by 12% sodium dodecyl sulfate-polyacrylamide gel electrophoresis (SDS-PAGE) and stained with Coomassie Blue R-250 (Biosharp, Beijing, China). The protein concentration was determined using the bicinchoninic acid (BCA) kit (Cowin, Jiangsu, China), and the purified protein was stored at −80°C.

The purified rLSDV-gap protein was subjected to SDS-PAGE and then transferred electrophoretically onto a polyvinylidene difluoride (PVDF) membrane (Millipore, Merck, Germany) for western blot assay. The membrane was blocked with 5% (w/v) skim milk in TBST buffer (TBS containing 0.05% (v/v) Tween-20) for 2 hr at 25°C. Then, the membrane was incubated with commercial monoclonal antibodies (Abbkine, Wuhan, China) targeting the six histidine tags which was diluted at a dilution ratio of 1 : 5,000 in advance for 12 hr at 4°C. After being washed for three times with TBST buffer, the membrane was incubated with commercial horseradish peroxidase (HRP)-goat antimouse IgG (H + L) (Abbkine, Wuhan, China) diluted at a dilution ratio of 1 : 5,000 for 1 hr at 25°C. After the incubation, the membrane underwent three additional washes with TBST buffer, and the rLSDV-gap protein band was visualized with ECL chemiluminescence reagents (Bio-Rad, Richmond, USA) by a chemiluminescence imaging system.

### 2.5. Evaluation of Differential Diagnostic Function of rLSDV-Gap Protein

Our previous research (data unpublished) has demonstrated the strong reactogenicity between rAXA19967.1 protein and LSDV or GTPV positive sera, and thus this study used rAXA19967.1 protein as positive control for evaluating the differential diagnostic function of rLSDV-gap protein. The concentrations of rAXA19967.1 protein and rLSDV-gap protein were determined individually using the BCA kit. Subsequently, antibody titers in the infected with LSDV and vaccinated with GTPV serum were assessed using rAXA19967.1 protein.

For western blot assay, equivalent amount rLSDV-gap protein and rAXA19967.1 protein were prepared. PDVF membranes were incubated with equivalent titer of infected and vaccinated serum antibody, respectively. Finally, western blot assay was performed to evaluate the differential diagnosis function of rLSDV-gap protein.

### 2.6. Establishment In-House Indirect ELISA (iELISA)

To establish in-house iELISA, in this study, a 96-well plate was coated with rLSDV-gap protein, and the optimum coating concentration was explored. The antigen was serially diluted in twofold increments ranging from 0.25 to 8 *μ*g/mL in a carbonate buffer solution (0.05 M CBS, pH 9.6). Subsequently, 100 *μ*L of rLSDV-gap protein solution at the same concentration was added to ELISA plates in every two columns incubated at 37°C for 2 hr, then transferred to 4°C stood for 14 hr. After the incubation, the plates were washed for three times with 300 *μ*L/well of PBS containing 0.1% (v/v) Tween-20 (PBST) with each wash lasting 3 min. The plates were then blocked with 200 *μ*L/well of blocking buffer (PBS containing 1% w/v fish gelatin) at 37°C for 1 hr. Subsequently, 100 *μ*L/well of LSDV-infected and GTPV-vaccinated serum samples were separately added to the plates in each column after serum sample was serially diluted in (1 : 100, 1 : 200, 1 : 400, 1 : 800, 1 : 1,600, 1 : 3,200, 1 : 6,400, and 1 : 12,800).

Then, rLSDV-gap protein was incubated under four different conditions, namely, 37°C for 1 hr, 37°C for 2 hr, 4°C for 16 hr, and 37°C for 2 hr, transferred to 4°C stood for 14 hr. In addition, rLSDV-gap protein was blocked at 37°C with various blocking reagents including 5% (w/v) skim milk, 2% (w/v) bovine serum albumin (BSA), and 1% (w/v) fish gelatin. Furthermore, the blocking solution was applied for different durations (1, 1.5, 2, 2.5, and 3 hr) at 37°C, and commercial rabbit antibovine IgG (H + L)/HRP was diluted to 1 : 4,000, 1 : 6,000, 1 : 8,000, and 1 : 10,000.

Finally, the chromogenic reaction was performed at 37°C for 5, 10, 15, and 20 min using the same method described above to determine the optimal chromogenic reaction time for rLSDV-gap-based iELISA.

The plates coated with rLSDV-gap protein were then incubated with serum samples at 37°C for 1 hr and washed for three times with PBST. The plates were added with rabbit antibovine IgG (H + L)/HRP (Solarbio, Beijing, China) diluted at 1 : 8,000 with PBS and added at 100 *μ*L/well, incubated for 1 hr at 37°C and washed with PBST for three times. Finally, the plates were added with 100 *μ*L/well of the substate 3,3′, 5,5′-tetramethylbenzidine dihydrochloride (TMB, Solarbio, Beijing, China)/H_2_O_2_ and incubated at 37°C for 10 min for the chromogenic reaction. The chromogenic reaction was terminated by adding 50 *μ*L/well of ELISA stop solution (Solarbio, Beijing, China). OD_450_ values were measured using a microplate reader (BMG LABTECH, Offenburg, Germany).

Thirty cattle serum samples at 60 days postvaccination with GTPV were detected by in-house iELISA. The mean and standard deviation (SD) of the cut-off value (*S*/*P*%) were calculated, and the mean ± 3 SD was used to determine the cut-off value.

For evaluating the analytical specificity of rLSDV-gap-based iELISA, positive sera for LSDV, BVDV, IBRV, *M. Tb*, *P. multocida*, and *M. bovis* were detected. Both infected and vaccinated sera were diluted in ratios from 1 : 100 to 1 : 12,800 to assess the analytical sensitivity, and OD_450_ values were measured.

To assess the stability of plates coated with rLSDV-gap protein, in-house iELISA plates were tested at 37°C for durations of 1, 3, 5, and 7 days. Following high-temperature test, serum samples including infected with LSDV and serum vaccinated with GTPV were detected to determine whether rLSDV-gap-based iELISA plates were damaged by high temperature.

Serum samples (*n* = 9) were detected using three batches of rLSDV-gap-based iELISA. OD_450_ values were measured to calculate the coefficient of variation (CV) for evaluating repeatability and reproducibility. Different serum samples (*n* = 9) were tested in triplicate with in-house iELISA within a single batch for the repeatability analysis. For the reproducibility analysis, different serum samples (*n* = 9) were tested by three independent batches of in-house iELISA.

### 2.7. Application of In-House iELISA

In this study, 10 were vaccinated with GTPV serum samples to evaluate the differential diagnostic ability of the in-house iELISA. Then, this in-house iELISA was applied to detect 200 clinical bovine serum samples antibodies either infected with LSDV or vaccinated with GTPV backgrounds.

### 2.8. Statistical Analysis

The statistical analysis of data was performed using GraphPad Prism 8.3.0 (GraphPad Software, San Diego, CA, USA). Optimization of reaction conditions for in-house iELISA was analyzed using Ordinary one-way ANOVA. The comparison of OD_450_ values between serum samples from cattle vaccinated with GTPV and infected with LSDV was analyzed using Student's *t*-test. The following notations indicate substantial differences between groups:  ^*∗*^*P* < 0.05,  ^*∗∗*^*P* < 0.01,  ^*∗∗∗*^*P* < 0.001, and  ^*∗∗∗∗*^*P* < 0.0001.

## 3. Results

### 3.1. Recombinant Protein Expression

**Figure 1:**
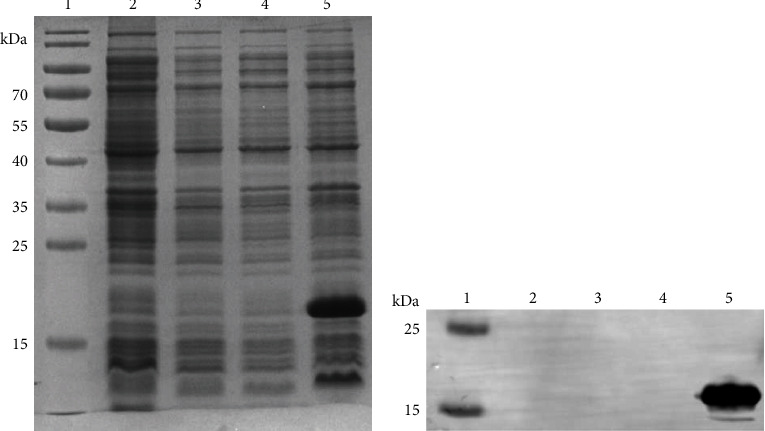
Identification of the expressed rLSDV-gap protein by SDS-PAGE and western blot assay. (a) SDS-PAGE of rLSDV-gap protein. (b) western blot assay of rLSDV-gap protein with the commercial monoclonal antibody to His-tag. Lane 1, molecular weight marker; Lane 2, pET-28a (+); Lane 3, pET-28a+IPTG; Lane 4, rLSDV-gap; and Lane 5, rLSDV-gap+IPTG.

The recombinant plasmid *rLSDV-gap* containing the fusion of six fragments was confirmed by PCR and DNA sequencing (data not shown). Subsequently, rLSDV-gap protein was successfully expressed in *E. coli* BL21 (DE3) and purified under denaturing conditions, with a molecular weight of 18 kDa (Figure 1(a)). Utilizing monoclonal antibody specific to the six histidine tags incubated the PVDF membrane, confirmed the successful expression of rLSDV-gap protein (Figure 1(b)).

### 3.2. Differential Diagnosis of rLSDV-Gap Protein

**Figure 2:**
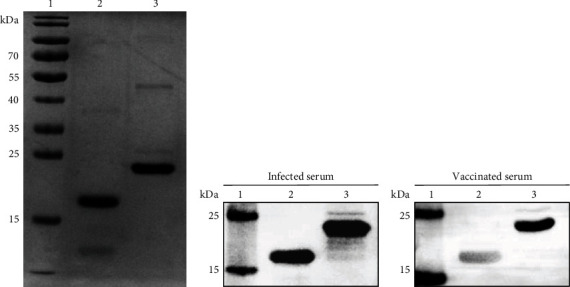
Differential diagnosis of rLSDV-gap protein. (a) SDS-PAGE of rLSDV-gap protein and rAXA19967.1 protein as the positive control. (b, c) Western blot assay of two proteins, respectively, with infected and vaccinated cattle serum. Lane 1, molecular weight marker; Lane 2, rLSDV-gap protein; and Lane 3, rAXA19967.1 protein.

The rLSDV-gap protein and rAXA19967.1 protein concentrations were 1.748 and 0.487 mg/mL, respectively. Twelve percentage SDS-PAGE result indicated that their molecular weights were 18 and 23 kDa, respectively, as expected (Figure 2(a)). To demonstrate the differential diagnostic function of rLSDV-gap protein, PVDF membranes were incubated with two differently diluted serum antibodies, one infected serum with a dilution of 1 : 800, and the other vaccinated serum with a dilution of 1 : 200. rAXA19967.1 protein was used as a positive control, LSDV-infected serum incubated with rLSDV-gap protein showed a clear and specific binding reaction, while GTPV-vaccinated serum incubated with rLSDV-gap protein showed very weak. These findings indicated that rLSDV-gap protein could be a differential diagnostic target (Figures 2(b) and 2(c)).

### 3.3. Optimization of the Concentrations of Antigen and Antisera

**Figure 3:**
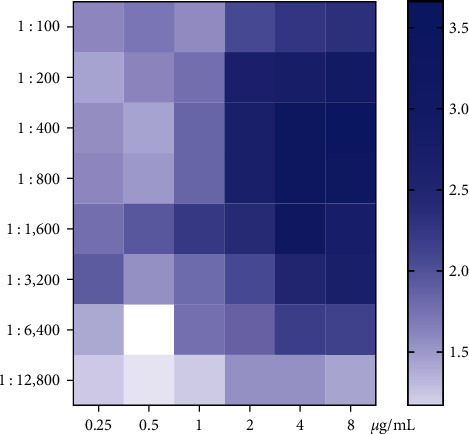
The concentrations optimization of antigen and antisera. The *X*-axis shows different concentrations of the antigen, while the left *Y*-axis shows different serum dilutions, and the right *Y*-axis represents *P*/*N* ratio; among then, *P* means OD_450_ value of infected serum, while *N* means OD_450_ value of vaccinated serum.

The optimal of rLSDV-gap protein as coating antigen concentrations, infected serum (*P*), and vaccinated serum (N) in iELISA was determined. The ratios of *P*/*N* OD_450_ values were calculated, and antigen and serum dilution concentrations were selected at the higher *P*/*N* ratio and the OD_450_ values in *P* close to 1.0. As a result, the optimal concentrations for antigen and serum were determined to be 4 *μ*g/mL, and a dilution of 1 : 800 of both *P* and *N* with a *P*/*N* ratio of 3.327 (Figure 3).

### 3.4. Development of iELISA Based on rLSDV-Gap Protein

**Figure 4:**
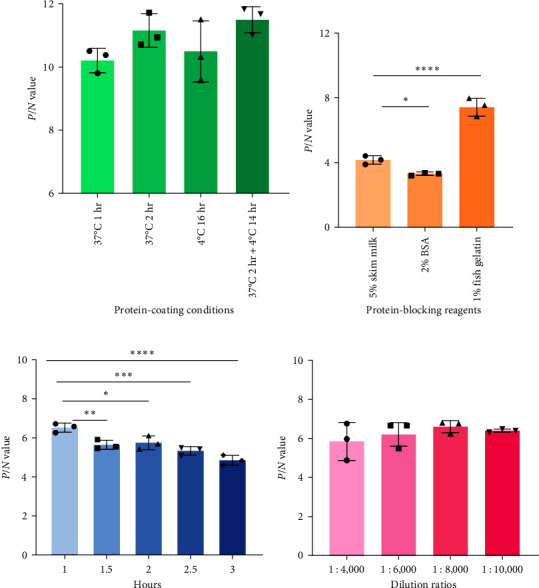
Optimization of reaction conditions for in-house iELISA. (a) Determination of optimal protein-coating conditions. (b) Determination of optimal protein-blocking reagents. (c) Determination of optimal blocking time by in-house iELISA. (d) Determination of optimal dilution of commercial rabbit antibovine IgG (H + L)/HRP. The *Y*-axis represents *P*/*N* value indicating the ratio of OD_450_ value between infected serum (*P*) and vaccinated serum (*N*) antibody responses as detected by in-house iELISA. Statistical significance was determined by ordinary one-way ANOVA,  ^*∗*^*P* < 0.05,  ^*∗∗*^*P* < 0.01,  ^*∗∗∗*^*P* < 0.001, and  ^*∗∗∗∗*^*P* < 0.0001.

*P*/*N* values were calculated separately for each condition. The data showed that the optimal condition was 37°C for 2 hr and transferred 4°C stood for 14 hr for coating rLSDV-gap protein (Figure 4(a)). The optimum blocking condition was determined to be in 1% (w/v) fish gelatin for 1 hr at 37°C (Figures 4(b) and 4(c)). The appropriate working dilution of rabbit antibovine IgG (H + L)/HRP was 1 : 8,000 (Figure 4(d)). The highest *P*/*N* ratio of OD_450_ value was 6.095, when TMB/H_2_O_2_ interaction time was optimized as 37°C for 10 min.

### 3.5. Determination of *S*/*P*% Value for In-House iELISA

The *S*/*P*% value was calculated by dividing the OD_450_ value of the vaccinated serum (*S*) by that of the positive control serum (*P*), infected serum as the above (*P*) with OD_450_ value of about 1.0. The mean *S*/*P*% value and standard deviation (SD) were 17.02 and 5.17, respectively. Furthermore, the cut-off value (mean + 2 SD) was calculated to be 27.36, and the value (mean + 3 SD) was calculated to be 32.53. Therefore, the *S*/*P*% value was 30% for in-house iELISA.

**Figure 5:**
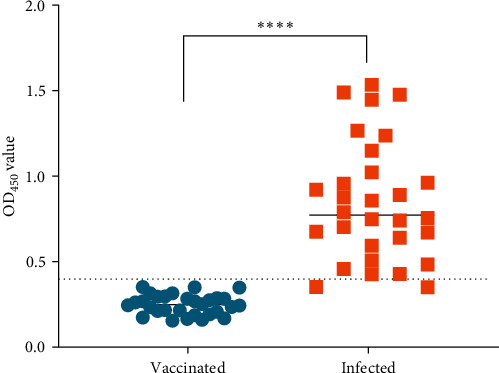
Detection of infected and vaccinated serum samples by in-house iELISA. The *X*-axis represents serum from vaccinated and infected cattle, while *Y*-axis represents OD_450_ value. Statistical significance was determined by Student's *t* test,  ^*∗∗∗∗*^*P* < 0.0001.

Using in-house iELISA to assess serum samples of both infected serum and vaccinated serum with known backgrounds, we observed that serum antibody levels in infected with LSDV cattle were significantly higher than those in vaccinated with GTPV cattle (*P* < 0.0001), and the cut-off value was 0.399 (Figure 5). Therefore, the test results were considered valid only if OD_450_ value of the positive control serum should be ≥0.399, while OD_450_ value of the negative control serum should be <0.399. The result showed the *S*/*P*% value derived from 30 serum samples from cattle vaccinated with GTPV and 30 serum samples from naturally infected with LSDV, serving as the criterion for in-house iELISA. The serum samples with *S*/*P*% value <30% were considered as negative, and those with *S*/*P*% value ≥30% were considered as positive.

### 3.6. Analytical Specificity and Sensitivity

The results showed no cross-reactions with positive sera for BVDV, IBRV, *M. Tb*, *P. multocida*, and *M. bovis*, except for specific reactions with LSDV-positive sera. When the infected serum was diluted to 1 : 6,400, the OD_450_ value was 0.412 (OD_450_ ≥ 0.399, considered positive). The lowest detection limit for positive serum was 1 : 6,400. The analytical sensitivity was substantially higher than dilution ratio of 1 : 800 specified by in-house iELISA.

### 3.7. Stability Test of In-House iELISA

The ELISA plates coated with the protein were placed at 37°C for 1, 3, 5, and 7 days. Afterward, they were utilized to measure OD_450_ values of both infected serum and vaccinated sera. The difference in OD_450_ values of serum samples within the same group was consistently less than 0.2 and within the valid range. As shown in Table 2, indicating that in-house iELISA could withstand 7-day high-temperature damage, exhibiting a good stability.

### 3.8. Repeatability and Reproducibility of In-House iELISA

The intra-assay CV ranged from 1.08% to 4.11%, all less than 5% (Table 3). Furthermore, the interassay CV was 0.00%–8.90%, all less than 10% (Table 4). These results indicated that in-house iELISA had good repeatability and reproducibility.

### 3.9. Validation of In-House iELISA in Differential Diagnosis

**Figure 6:**
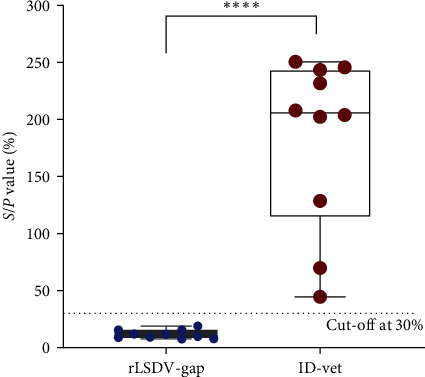
Validation of in-house iELISA in differential diagnosis. Ten vaccinated serum samples were tested with in-house iELISA, and the same serum samples were tested by ID Screen® Capripox Double Antigen Multi-species test kit. The *Y*-axis on the left was *S*/*P* value (%), and the *X*-axis represented two different detection methods. Statistical significance was determined by Student's *t* test,  ^*∗∗∗∗*^*P* < 0.0001.

When utilizing in-house iELISA to test 10 vaccinated serum samples that were positive for both serum antibodies and neutralizing antibodies. As shown in Figure 6, all serum samples tested negative by rLSDV-gap-based iELISA. However, the results indicated the *S*/*P*% value of the same vaccinated serum samples when detected by ID Screen® Capripox Double Antigen Multi-species test kit (ID-Vet, Grabels, France), with samples having *S*/*P*% ≥ 30% considered positive. In summary, rLSDV-gap-based iELISA demonstrated excellent differential diagnosis capability.

### 3.10. Application of In-House iELISA

Using in-house iELISA based on rLSDV-gap protein, we detected serum antibodies in 200 clinical serum samples. In the vaccinated herd, there were no positive samples (0/141) indicating the strong differentiation ability of this method. On the other hand, in the infected herds, the overall positivity was 33.90% (20/59) (95% CI: 22.08–47.39) varied with different herds, the beef herd 68.42% (13/19) (95% CI: 43.45–87.42), and the yak herd 17.50% (7/40) (95% CI: 7.34–32.78).

## 4. Discussion

WOAH recommends virus neutralization test (VNT) as the gold standard of LSD diagnosis [15]. However, ELISA has been developed as a diagnostic tool for evaluating the immune response to LSDV by several investigators likely because ELISA is simple and rapid to get test results [22, 23]. Samojlović et al. [22] utilized a commercial ELISA kit to detect LSDV serum antibodies and matched the kit's performance to VNT. The results suggest that the performance of the ELISA diagnostic kit is as effective as the gold standard, highlighting the importance of the commercial ELISA kit as a serological tool for detecting LSDV serum antibodies [24, 25]. However, there are no ELISA kit as a differentiating diagnostic tool for lumpy skin disease. Hence, we developed an in-house iELISA to detect serum samples naturally infected with LSDV. Due to virus neutralization test being the gold standard, we took 10 vaccinated serum samples with higher antibody titer and neutralizing antibodies to confirm the differential diagnostic function of rLSDV-gap protein. As expected, our method tested the vaccinated serum samples to be negative, showing the potential of rLSDV-gap protein as an excellent DIVA target. Thirty vaccinated sera were utilized to determine the cut-off value in rLSDV-gap-based iELISA, but the number of vaccinated serum samples employed in establishing this method was limited.

The synthesized gene originated from unique fragments of LSDV, and we inserted five “GSGS” linker flexible peptide between six fragments. This strategy ensured the free stretch of these six fragments. Although the mosaic protein is of viral origin, it can be expressed well in *E. coli* system. As shown in Figure S1(a), we confirmed the selected six fragments of *rLSDV-gap* were highly conservative with other isolated strains. However, there were 88 nucleotides of similarity between *rLSDV-gap* and the nonprotein coding region of GTPV AV41 strain (Figure S1(c)). To remove this cross-reaction, we optimized in-house iELISA conditions such as increasing serum dilution to 1 : 800, theoretically ensuring that rLSDV-gap protein had a good specificity.

Then, we did positive sera with *M*. *bovis*, *P*. *multocida*, *M*. *Tb*, BVDV, and IBRV separately incubation with rLSDV-gap protein to confirm the excellent specificity of the purified antigen by western blot assay (Figure S2). The reaction between rLSDV-gap protein and LSDV positive sera confirmed its activity and specificity, rendering it suitable for the establishment of serological diagnosis for LSD. Furthermore, this protein-coated plates kept for 12 months at 4°C, and did not significant decrease in OD_450_ value when used to test reference serum samples indicating its good stability (Figure S3). Overall, rLSDV-gap-based iELISA had an excellent differential diagnostic value for areas using GTPV vaccine to prevent and control LSDV outbreaks. In the future, it would be better to test more cattle serum samples using rLSDV-gap-based iELISA to improve its applicability more accurately.

In conclusion, we designed and synthesized a gene including six fragments of the nucleotide sequences of protein-coding region unique to LSDV rather than GTPV, and rLSDV-gap protein as a DIVA antigen in the form of in-house iELISA can effectively distinguish serum antibodies in cattle naturally infected with LSDV from those vaccinated with GTPV.

## Figures and Tables

**Table 1:** The sequences of rLSDV-gap.

The sequences of rLSDV-gap	
Nucleotide sequence (5′–3′)	ATG AAC AAT TGT AAT GAA ATT GAT GAA AAC GGT AGC GGT AGC ATG CAT CAG ATG AAT CTT ACA ACA GCA ATG CCT AAC AAT AAC GCC TTT AGC ATA GGA ACT GTA CTA ATT TTG GGT AGC GGT AGC ATG GGT GAT GGT GAT GGT AAT GGT GCT AAT GAT AGT AAT GGT AGC GGT AGC ATG AAA AAA AAA AAT TTT TTA GTT TTA AAT ACA ACA AAA ATA AAA GGT AGC GGT AGC ATG GAT AAT ACT AAC GAC TAT GAT GAA GGT AGC GGT AGC ATG GAT ACA TGT AGT TAT TGG AAT ACA ATA CCG TTA GAA ATA AAA TTT AAA ATA GTA AAT AAT TTA TCA CTT AAT GAC ATA GAA ATG TTT TTG AAA AAT AAT AAA AAA

Amino acid sequence (NH_2_– to COOH–)	MNNCNEIDENGSGSMHQMNLTTAMPNNNAFSIGTVLILGSGSMGDGDGNGANDSNGSGSMKKKNFLVLNTTKIKGSGSMDNTNDYDEGSGSMDTCSYWNTIPLEIKFKIVNNLSLNDIEMFLKNNKK

**Table 2:** Stability test of in-house iELISA.

Sera	Days at 37°C			
	1	3	5	7
Infected	1.088	1.028	1.051	1.058
Vaccinated	0.294	0.242	0.247	0.298
*S*/*P*%	27.022	23.541	23.501	28.166

**Table 3:** Repeatability of in-house iELISA.

Reference serum	Repeatability (OD_450_ value)			Coefficient of variation, CV (%)
	Test 1	Test 2	Test 3	
1 (Infected)	0.928	0.974	0.929	2.784
2 (Infected)	0.867	0.877	0.886	1.084
3 (Infected)	0.900	0.907	0.929	1.659
4 (Vaccinated)	0.298	0.279	0.279	3.845
5 (Vaccinated)	0.204	0.188	0.198	4.110
6 (Vaccinated)	0.198	0.192	0.204	3.030
7 (Negative)	0.122	0.126	0.119	2.871
8 (Negative)	0.154	0.146	0.152	2.763
9 (Negative)	0.155	0.160	0.162	2.268

**Table 4:** Reproducibility of in-house iELISA.

Reference serum	Reproducibility, CV (%)		
	Test 1	Test 2	Test 3
1 (Infected)	2.784	3.987	3.721
2 (Infected)	1.084	5.025	3.403
3 (Infected)	1.659	2.304	0.323
4 (Vaccinated)	3.845	8.904	1.845
5 (Vaccinated)	4.110	0.000	8.383
6 (Vaccinated)	3.030	4.044	3.375
7 (Negative)	2.871	5.184	4.710
8 (Negative)	2.763	5.177	0.806
9 (Negative)	2.268	4.751	2.785

## Data Availability

All data generated and analyzed during this study are included in the main manuscript or supplementary materials/files.

## Supplementary Materials

Supplementary Materials Figure S1: the specificity of nucleotide sequences of rLSDV-gap was analyzed by Blast analysis. Figure S2: analysis of the specificity of rLSDV-gap protein by western blot assay. Figure S3: the results of rLSDV-gap based iELISA plates stored at 4°C. Table S1: the results of serum neutralizing antibody titers of vaccinated with GTPV AV41. Table S2: the results of serum neutralizing antibody titers of naturally infected with LSDV. Table S3: the results of vaccinated sera tested by ID Screen® Capripox Double Antigen Multi-species test kit. Table S4: the results of infected sera tested by ID Screen® Capripox Double Antigen Multi-species test kit.
