# Supplementary material for: Development of a Synthesized Gene Unique to Lumpy Skin Disease Virus and Its Application in Serological Differentiation of Naturally Infected from Vaccinated Cattle with Attenuated Goat Pox Vaccine
Source: Transbound Emerg Dis. 2024 Jun 10;2024:7800855. doi: 10.1155/2024/7800855 (PMC12017463; doi:10.1155/2024/7800855)
Supplement: Supplementary Materials — Figure S1: the specificity of nucleotide sequences of rLSDV-gap was analyzed by Blast analysis. Figure S2: analysis of the specificity of rLSDV-gap protein by western blot assay. Figure S3: the results of rLSDV-gap based iELISA plates stored at 4°C. Table S1: the results of serum neutralizing antibody titers of vaccinated with GTPV AV41. Table S2: the results of serum neutralizing antibody titers of naturally infected with LSDV. Table S3: the results of vaccinated sera tested by ID Screen® Capripox Double Antigen Multi-species test kit. Table S4: the results of infected sera tested by ID Screen® Capripox Double Antigen Multi-species test kit. [file 7800855.f1.doc]

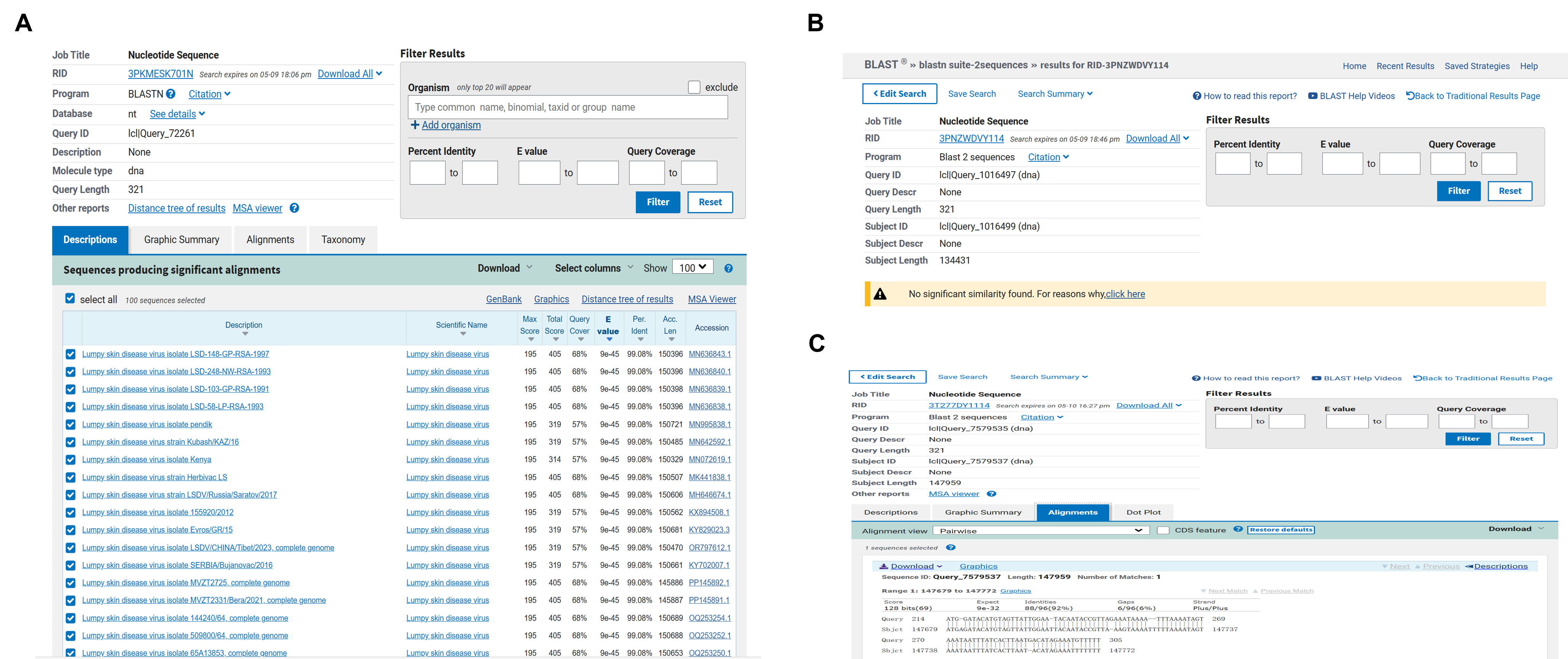


Supplementary Fig.1 The specificity of nucleotide sequences of *rLSDV-gap* was analyzed by Blast analysis. Supplementary Fig.1A The similarity of *rLSDV-gap* at nucleotide level with other publicized LSDV sequences in GenBank by online Blast analysis. The nucleotide sequence of *rLSDV-gap* was aligned by Blast with publicized 100 sequences in GenBank and the results showed that *rLSDV-gap* was very conservative having a 99.08% similarity with other isolated strains. Supplementary Fig.1B The similarity of *rLSDV-gap* at nucleotide level with the genome of bovine papular stomatitis virus in GenBank by online Blast analysis, and there was no similarity between the synthesized *rLSDV-gap* and the genome of bovine papular stomatitis virus. Supplementary Fig.1C The similarity of *rLSDV-gap* at nucleotide level with GTPV AV41 in GenBank by online Blast analysis, matched 88/96 nucleotides in the non-protein coding region on GTPV AV41.


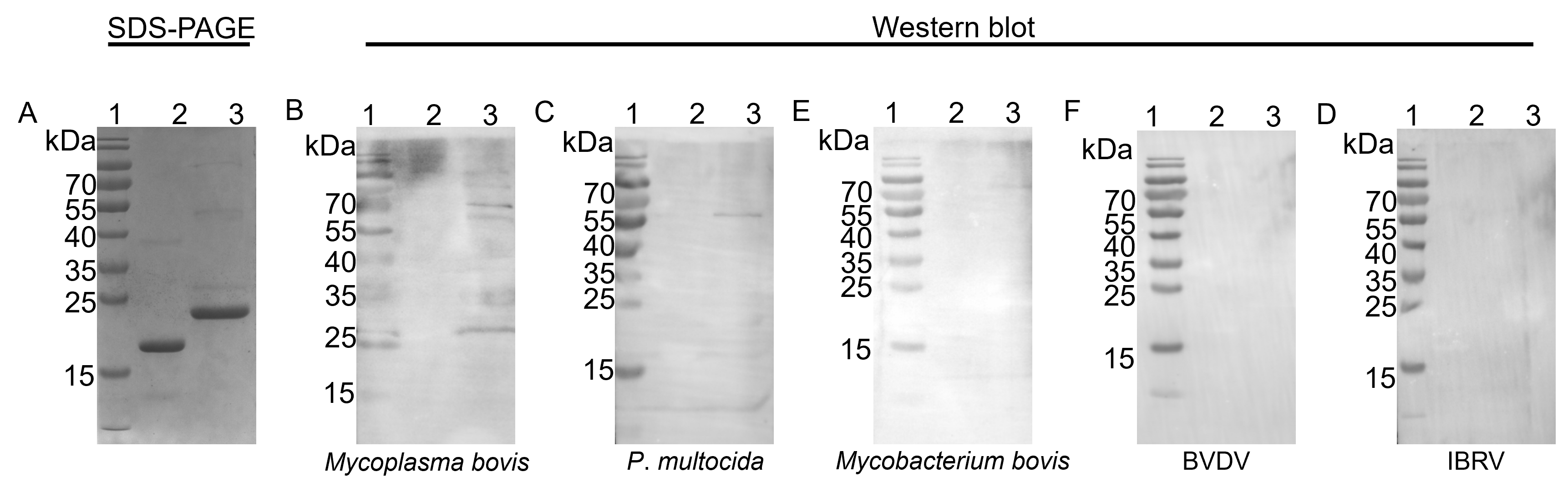


Supplementary Fig.2 Analysis of the specificity of rLSDV-gap protein by western blot assay. Positive sera with *Mycoplasma bovis*, *P*. *multocida*, *Mycobacterium bovis*, BVDV, and IBRV separately incubation with rLSDV-gap protein to confirm the excellent specificity of the purified antigen by western blot assay. Lane 1, molecular weight marker; Lane 2, rLSDV-gap protein; Lane 3, rAXA19967.1 protein.


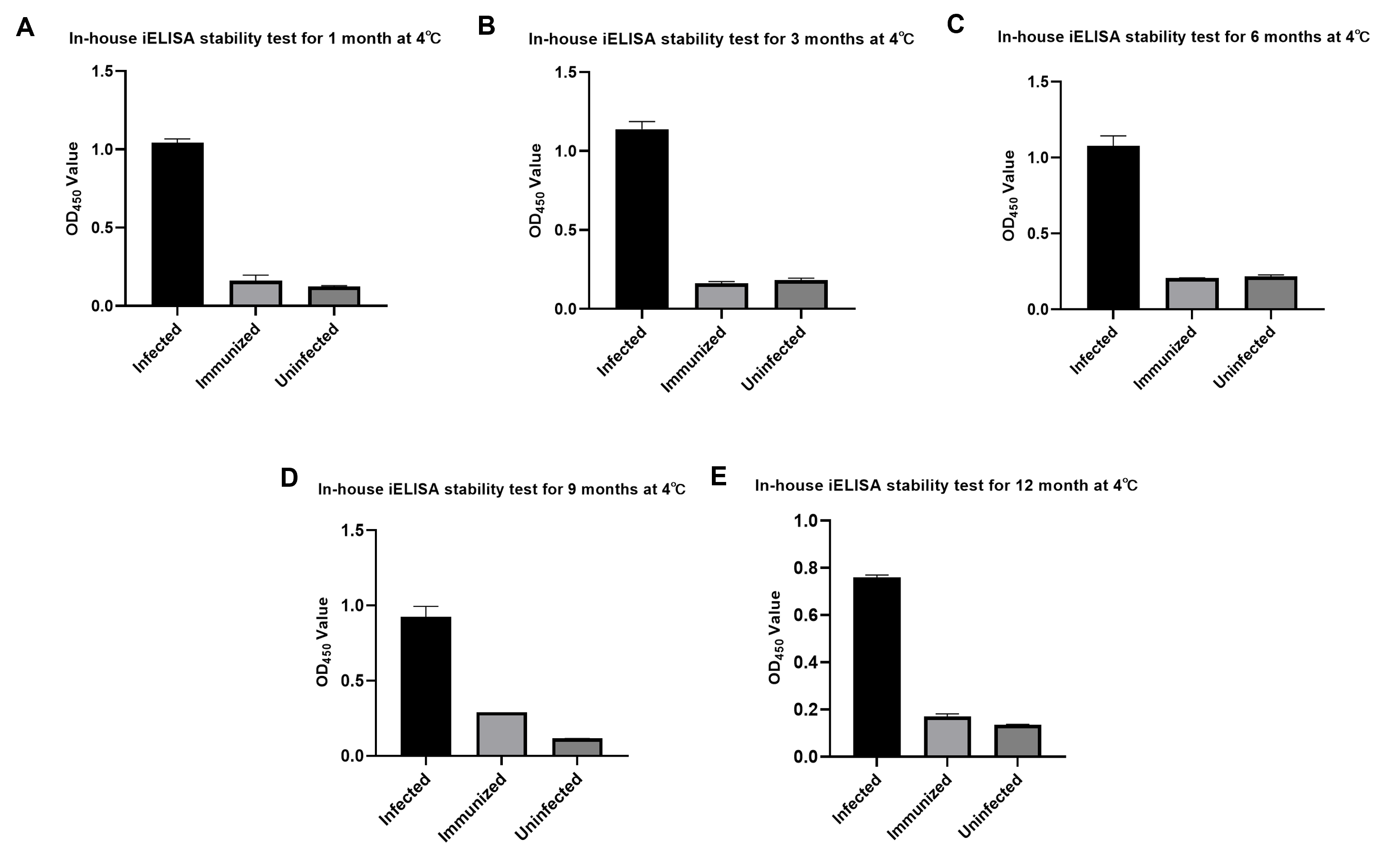


Supplementary Fig.3 The results of rLSDV-gap based iELISA plates stored at 4℃. The rLSDV-gap protein coated plates kept for 1, 3, 6, 9, and 12 months. The rLSDV-gap protein coated plates kept for 12 months at 4℃, and didn't significant decrease of OD_450_ value when used to test reference serum samples indicating its good stability.

Supplementary Table 1 Serum neutralizing antibody titers of vaccinated with GTPV AV41

| Cattle ID | Neutralizing antibody titer level | Cattle ID | Neutralizing antibody titer level |
| --- | --- | --- | --- |
| 1 | + (< 1: 80) | 16 | + (< 1: 40) |
| 2 | + (1: 40) | 17 | + (1: 10) |
| 3 | + (1: 40) | 18 | - (< 1: 5) |
| 4 | + (1: 160) | 19 | + (1: 40) |
| 5 | + (1: 20) | 20 | + (< 1: 80) |
| 6 | + (1: 40) | 21 | + (1: 160) |
| 7 | + (1: 10) | 22 | + (1: 20) |
| 8 | + (1: 40) | 23 | + (1: 40) |
| 9 | + (< 1: 80) | 24 | + (1: 80) |
| 10 | + (1: 80) | 25 | + (< 1: 160) |
| 11 | + (1: 80) | 26 | + (1: 40) |
| 12 | + (1: 160) | 27 | + (1: 40) |
| 13 | + (1: 10) | 28 | + (< 1: 40) |
| 14 | + (1: 160) | 29 | + (1: 20) |
| 15 | + (< 1: 160) | 30 | + (1: 160) |

Note: "-" represented that the neutralizing antibody titer was negative (< 1: 5); "+" represented that the neutralizing antibody titer was positive (≥ 1: 5).

Supplementary Table 2 Serum neutralizing antibody titers of naturally infected with LSDV

| Cattle ID | Neutralizing  antibody titerlevel | Cattle ID | Neutralizing  antibody titer level |
| --- | --- | --- | --- |
| 1 | + (1: 80) | 16 | + (1: 320) |
| 2 | + (1: 160) | 17 | + (1: 40) |
| 3 | + (1: 80) | 18 | + (1: 80) |
| 4 | + (1: 80) | 19 | + (1: 40) |
| 5 | + (1: 160) | 20 | + (1: 80) |
| 6 | + (1: 160) | 21 | + (1: 160) |
| 7 | + (1: 20) | 22 | + (1: 160) |
| 8 | + (1: 20) | 23 | + (1: 40) |
| 9 | + (1: 40) | 24 | + (1: 80) |
| 10 | + (1: 40) | 25 | + (1: 80) |
| 11 | + (1: 40) | 26 | + (1: 320) |
| 12 | + (1: 40) | 27 | + (1: 320) |
| 13 | + (1: 40) | 28 | + (1: 320) |
| 14 | + (1: 40) | 29 | + (1: 40) |
| 15 | + (1: 40) | 30 | + (1: 80) |

Note: "+" represented that the neutralizing antibody titer was positive (≥ 1: 5).

Supplementary Table 3 Vaccinated sera were tested by ID Screen® Capripox Double Antigen Multi-species test kit

| Cattle ID | S/P% | Cattle ID | S/P% |
| --- | --- | --- | --- |
| 1 | + (74.58) | 16 | + (400.63) |
| 2 | + (101.43) | 17 | + (63.07) |
| 3 | + (46.28) | 18 | + (41.52) |
| 4 | + (310.04) | 19 | + (67.19) |
| 5 | + (43.68) | 20 | + (425.99) |
| 6 | + (122.82) | 21 | + (457.21) |
| 7 | + (169.36) | 22 | + (277.18) |
| 8 | + (354.89) | 23 | + (193.19) |
| 9 | + (31.42) | 24 | + (393.19) |
| 10 | + (37.29) | 25 | + (71.16) |
| 11 | + (211.86) | 26 | + (383.04) |
| 12 | + (358.54) | 27 | + (50.40) |
| 13 | + (60.37) | 28 | + (167.67) |
| 14 | + (231.81) | 29 | + (62.76) |
| 15 | + (184.47) | 30 | + (217.12) |

Note: S/P% < 30%, considered negative (-); S/P% ≥ 30%, considered positive (+).

Supplementary Table 4 Infected sera were tested by ID Screen® Capripox Double Antigen Multi-species test kit

| Cattle ID | S/P% | Cattle ID | S/P% |
| --- | --- | --- | --- |
| 1 | + (32.10) | 16 | + (143.32) |
| 2 | + (47.08) | 17 | + (124.88) |
| 3 | + (123.51) | 18 | + (161.44) |
| 4 | + (172.44) | 19 | + (50.24) |
| 5 | + (73.06) | 20 | + (36.00) |
| 6 | + (255.74) | 21 | + (195.39) |
| 7 | - (12.85) | 22 | + (178.72) |
| 8 | - (3.37) | 23 | + (138.75) |
| 9 | + (106.05) | 24 | + (159.14) |
| 10 | + (116.06) | 25 | + (62.06) |
| 11 | + (96.04) | 26 | + (117.56) |
| 12 | + (122.27) | 27 | + (159.00) |
| 13 | + (103.44) | 28 | + (143.80) |
| 14 | + (136.29) | 29 | + (177.50) |
| 15 | + (146.32) | 30 | + (185.10) |

Note: S/P% < 30%, considered negative (-); S/P% ≥ 30%, considered positive (+).
